# Pupil dilation response elicited by violations of auditory regularities is a promising but challenging approach to probe consciousness at the bedside

**DOI:** 10.1038/s41598-023-47806-1

**Published:** 2023-11-21

**Authors:** Aude Sangare, Marion Quirins, Clémence Marois, Mélanie Valente, Nicolas Weiss, Pauline Perez, Amina Ben Salah, Esteban Munoz-Musat, Sophie Demeret, Benjamin Rohaut, Jacobo D. Sitt, Cecile Eymond, Lionel Naccache

**Affiliations:** 1 Assistance Publique – Hôpitaux de Paris, Groupe Hospitalier Pitié-Salpêtrière, Charles Foix, Département de Neurophysiologie, Sorbonne Université, Paris, France; 2 INSERM U 1127, PICNIC, Lab, Institut du Cerveau et de la Moelle Épinière, ICM, 75013 Paris, France; 3 Département de Neurologie, Hôpital Fondation Adolphe de Rothschild, Paris, France; 4 AP-HP.Sorbonne Université, Hôpital Pitié-Salpêtrière, Département de Neurologie, Unité de Médecine Intensive et Réanimation à Orientation Neurologique & Groupe de Recherche Clinique en REanimation et Soins Intensifs du Patient en Insuffisance Respiratoire aiguE (GRC-RESPIRE) Sorbonne Université, Sorbonne Université, Paris, France; 5 Brain Liver Pitié-Salpêtrière (BLIPS) Study Group, INSERM UMR_S 938, Centre de Recherche Saint-Antoine (CRSA), Maladies Métaboliques, Biliaires et Fibro-Inflammatoire du Foie & Institute of Cardiometabolism and Nutrition (ICAN), Paris, France; 6 Anesthesia and Intensive Care Unit, Lyon Medical Intensive Care Unit, Edouard, Herriot Hospital, Hospices Civils de Lyon, 69437 Lyon, France

**Keywords:** Neuroscience, Medical research, Neurology

## Abstract

Pupil dilation response (PDR) has been proposed as a physiological marker of conscious access to a stimulus or its attributes, such as novelty. In a previous study on healthy volunteers, we adapted the auditory “local global” paradigm and showed that violations of global regularity elicited a PDR. Notably without instructions, this global effect was present only in participants who could consciously report violations of global regularities. In the present study, we used a similar approach in 24 non-communicating patients affected with a Disorder of Consciousness (DoC) and compared PDR to ERPs regarding diagnostic and prognostic performance. At the group level, global effect could not be detected in DoC patients. At the individual level, the only patient with a PDR global effect was in a MCS and recovered consciousness at 6 months. Contrasting the most regular trials to the most irregular ones improved PDR’s diagnostic and prognostic power in DoC patients. Pupillometry is a promising tool but requires several methodological improvements to enhance the signal-to-noise ratio and make it more robust for probing consciousness and cognition in DoC patients.

## Introduction

Probing residual cognition in patients with disorders of consciousness (DoC) is of tremendous importance to provide an accurate neurologic prognosis and to choose appropriate therapeutics^1^. These states include in particular the vegetative state (VS), also termed unresponsive wakefulness syndrome (UWS), and the minimally conscious state (MCS). To diagnose DoC with the best precision, a cumulative set of evidence converges to the necessity to complete a rich, rigorous and repeated behavioural examination with additional measures of brain activity^2–6^. In this perspective, developments of various indirect physiological measures of brain activity appear as potentially relevant tools^7–9^. One of these promising tracks of clinical research is pupillometry. Indeed, pupillometry is more easily accessible at the bedside, does not require complex data processing such as EEG-based measures, and could provide an objective physiological measure of residual cortically mediated behaviours^10^. At the acute phase, pupillary light reactivity is a well-described prognostic factor after severe brain damages^11–14^. More recently, pupil dilation response (PDR) has been used in intensive care unit (ICU) to monitor nociception^15^, communicate^16^ and demonstrate command-following^16,17^.

Previous studies conducted on healthy subjects suggested that PDR may be used as a somatic signature of cognitive effort^18^ and conscious access^19–23^. Conscious access refers to a cognitive stage of stimulus processing defined by the ability of the individual to self-report (verbally or not) stimulus presence or some of its attributes^6,24,25^. Pupil diameter increases before perceptual reports of ambiguous stimuli^20,21^, or along with the recognition stage when presented with ambiguous transforming stimuli^22,26^. PDR is also modulated by subjective image interpretation of brightness, motion or distance^19,23,27,28^. Moreover, PDR has been reliably identified as a physiological marker of standard vs deviant auditory stimulus discrimination in oddball paradigms with various manipulations of the stimulus probability, property, and intensity^29,30^.

In a previous study on healthy volunteers, we adapted to pupillometric recordings the auditory “local global” paradigm we previously conceived and designed for ERPs. In the “local global” paradigm^31^, two types of auditory regularities are crossed: an intra-trial regularity (local effect) and an inter-trial regularity (global effect). High-density scalp EEG, intracranial recordings and fMRI revealed that violation of the local regularity (local effect) was associated with a mismatch negativity (MMN) response originating from auditory cortices, whereas violation of global regularity (global effect) was associated with a P3b response originating from a widespread cortical network including prefrontal and parietal cortices, anterior cingulate cortex and hippocampal structures^31,32^. Crucially, in the passive attentive version of the local–global paradigm, a global effect (P3b) was present only in individuals who were spontaneously aware of the existence of a global regularity, and the global effect vanished when conscious resources were allocated to a concurrent rapid serial visual presentation task^31^.

In DoC patients, a global effect was observed only in conscious individuals (emergence from MCS) or in individuals who subsequently rapidly regained consciousness, suggesting that they were actually covertly conscious during the local–global task^31,33–35^. This notion of covert consciousness in behavioral unresponsive patients is often referred to the generic name of Cognitive-Motor-Dissociation (CMD) and may actually concern a significant fraction of acute DoC patients^36,37^.

It is essential to remind the predicted asymmetry prevailing between global effect and conscious state. We showed that individuals with a global effect consciously accessed to stimulus global regularity status (*i.e.,* global deviant or global standard), and were therefore in a conscious state. This prediction was tested and confirmed in a large set of studies using various brain activity measures (EEG, MEG, iEEG, fMRI, PDR) combined with subjective reports^31,32,38^. So, as a clear consequence, if global effect is a specific measure of consciousness, its sensitivity is very limited given that it is not enough to be in a conscious state. Individuals also have to pay attention to the task, hear stimuli, be motivated, stay awake etc. In a recent study conducted on 236 DoC patients, 28% of patients had an ERP global effect^33^ (presence of global effect predicted behaviorally overt consciousness recovery in survivors with high specificity (Sp = 84%) and high positive predictive value (PPV = 80%) but with low sensitivity (Se = 35%) and low negative predictive value (NPV = 42%). Indeed, in the absence of any global effect, a patient may still be conscious but unable to understand the instructions, actively maintain attention, deploy working memory processes or may just be distracted.

We adapted the ”local–global” task to the slow dynamics of PDR and used it in two substantial sets of healthy volunteers with both an acoustic and a phonemic version of this task^39^. In a set of experiments, we could transpose ERPs results to PDR the following way : (i) we showed the existence of a generic PDR to any kind of auditory stimulus (local standard/deviant; global standard/deviant); (ii) this generic PDR was not affected by the local effect (*i.e.* similar PDR for local standard and local deviant trials); (iii) the global effect was associated to a significant PDR (*i.e.* larger PDR for global deviant than for global standard trials); (iv) this PDR global effect was associated to conscious access to violations of global regularity. This global effect was present in active versions of the task (*i.e.,* subjects were explicitly instructed about global deviance and counted global deviant trials). In passive attentive versions of the task, global effect was present only in individuals who spontaneously and consciously discovered the existence of a global rule. All these results suggested that a global effect PDR should be a relevant sign of conscious processing.

In the present study, we aimed to test this key prediction,—as well as more generally the relevance of auditory PDR in DoC patients -, to improve both diagnosis and prognosis performance.

## Materials and methods

### Patients

From 27/08/2013 to 02/06/2015, 42 patients referred to the Neurology Intensive Care Unit of the Pitié-Salpêtrière University hospital (APHP, Sorbonne University, Paris, France) for an evaluation of consciousness were screened for participation in the study. All behavioural procedures performed in the present study were performed in accordance with the ethical standards of the Helsinki declaration (1964) and its later amendments and were approved by the local ethical committee (Comité de protection des personnes Ile de France I, #2013-A00106-39). An informed consent was obtained from all the participants’ legal guardians for both study participation and publication of identifying images in an online open-access publication.

### Behavioral examination

Clinical examination was performed by an experienced neurologist and included the Coma Recovery Scale Revised (CRS-R)^40^ and the assessment of brainstem reflexes. Of particular interest here, the integrity of the pupillary light reflex of each eye was checked. Comatose patients with an arousal Coma Recovery Scale subscale inferior to 1 were excluded, as well as conscious patients (EMCS scoring). An auditory startle was defined as present if eyelid flutter or blink occurs immediately following an auditory stimulus on at least two trials, as previously described^41^. Crucially, a CRS-r score was performed immediately before pupillometric recordings.

Clinical outcome at 6 months and 1 year after the initial clinical evaluation was assessed using the Glasgow Outcome Scale-Extended (GOSE) and items of the CRS-R through a phone interview with a close relative or with patients’ physicians or were extracted from medical records.

### Auditory paradigm and procedure

**Figure 1:**
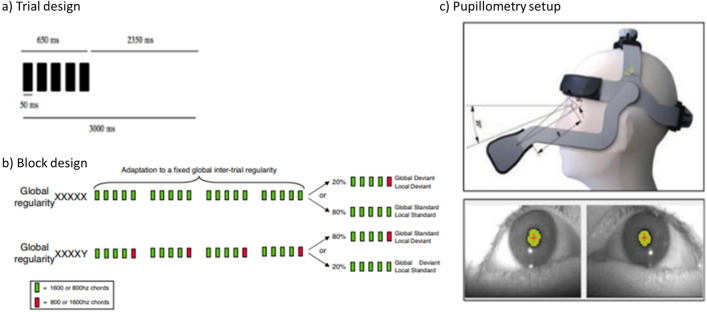
Auditory paradigm and pupillometry setup Reproduced with permission from^39^ (**a**) On each trial 5 sounds were presented. (**b**) Each of the four experimental blocks started with four identical series of sounds defining the global regularity, before delivering global standard (80%) or global deviant (20%) trials, that were also either local standard or local deviant trials. (**c**) Eyebrain mobile device including two independent infrared cameras centred on each pupil captures pupil images of each eye at a sampling rate of 120 Hz.

We used a version of the classical auditory ”local global” paradigm^31^ that was previously adapted to the slower temporal dynamic of pupil variations and validated in healthy volunteers^39^. This auditory paradigm crosses two types of auditory regularities (Fig. 1): an intra-trial regularity (local effect) and an inter-trial regularity (global effect). Repeated series of five sounds were presented via the laptop speakers. Each sound lasted 50 ms (SOA 150 ms) and could be either a low-pitched tone (A) or a high-pitched tone (B).

Four different series were used, the first two consisted of the same five sounds to conserve the local regularity (either AAAAA or BBBBB, called local standard (LS) stimulus); and the other two with the final sound swapped to break the local regularity (either AAAAB or BBBBA, called local deviant (LD) stimulus). The global regularity was defined by the repetition of one of the series in about 80% of the trials (global standard (GS) or frequent stimulus). This global regularity was broken in about 20% of the trials by the presentation of an alternative series with a different local regularity (global deviant (GD) or infrequent stimulus). Finally, we used four block types by experiment designed by the frequent sequence (global standard): type AAAAA (AAAAA frequent is LSGS / AAAAB infrequent is LDGD), type BBBBB (BBBBB frequent is LSGS /BBBBA infrequent is LDGD), type AAAAB (AAAAB frequent is LDGS / AAAAA infrequent is LSGD), type BBBBA (BBBBA frequent is LDGS / BBBBB infrequent is LSGD). Each block started with five identical series of the frequent type to define the global regularity. The number of infrequent stimuli per block was randomized between 4 and 7 and each one was followed by a frequent stimulus to remind the global regularity. The time interval between the two series was 3000 ms (instead of 1350–1650 ms in evoked potentials) in order to adapt to the slower dynamics of pupil dilation/constriction. Each block included 30 trials and lasted 90 s (in total the experience lasted 6 min).

### Pupillometry: data acquisition and analysis

Pupillometric data were recorded binocularly at a 120 Hz sampling rate with the Mobile eyetracked (e(ye)BRAIN, www.eye-brain.com, France) that received CE marking approval for medical purposes. The acquisition was performed at the bedside, in intensive care units where the luminance and environment noise level were not controlled. Auditory stimuli were delivered by the MeyeParadigm software and raw data were stored and extracted by MeyeAnalysis software (www.eye-brain.com, France).

Raw pupil data were first pre-processed with the ET-remove-artifacts toolbox (https://github.com/EmotionCognitionLab/ET-remove-artifacts). Dilation speed outliers and eye blinks were detected and removed, and a linear interpolation was used to fill in gaps in the data. Then, these pre-processed pupil data were imported into the Brainstorm toolbox (available for download online under the GNU general public license (http://neuroimage.usc.edu/brainstorm)^42^). Data were filtered with a 10 Hz low pass filter, and epochs for each trial were extracted from 200 to 3000 ms after the beginning of the first sound. A trial was rejected when more than 25% of the data was interpolated. A patient dataset was excluded if it included more than 50% of rejected trials. All patients with a trial rejection rate superior to 25% (but inferior to 50%) were labeled as “poor quality” datasets. When the clinical examination revealed an asymmetric pupillary reflex, data from the most reactive eye were analysed. Otherwise, both eyes were analysed and we kept the best quality dataset (i.e., left or right eye dataset) for further analyses.

### Event-related potentials

In complement to pupillometry, ERP were acquired during the auditory “local–global” paradigm in a different session at the bedside. ERPs were recorded at 250 Hz with a 256-electrodes geodesic sensor net (EGI R, Oregon, USA) referenced to the vertex. Trials were band-pass filtered (0.5–45 Hz), then segmented in epochs ranging from − 200 to + 1.344 ms from the first sound onset. Electrodes with voltages exceeding 100 µV in more than 50% of the epochs were removed. Moreover, voltage variance was computed across all correct electrodes. Electrodes with a voltage variance Z-score higher than four were removed. This process was repeated four times. Bad electrodes were interpolated using a spline method. Epochs were labeled as bad and discarded when voltage exceeded 100 µV in more than 10% of electrodes. Moreover, voltage variance was computed across all correct epochs, and epochs with a Z-score larger than four were removed. This process was also repeated four times. The remaining stimulus-locked epochs were averaged and digitally transformed to an average reference. An 800 ms baseline correction (before the onset of the fifth sound of the trial) was applied. EEG recordings had to satisfy the following two criteria to be considered of good quality: they should include a minimum of 75% valid channels and 30% of valid epochs, otherwise they were considered to be of “poor quality”. Preprocessing was implemented using MNE Python environment. Similarly to pupillometric recordings, a CRS-R was scored immediately before EEG acquisition.

### Statistics

#### Population characteristics

Population characteristics (age, sex, time since brain injury, etiology, medication affecting pupil size) between VS/UWS and MCS were compared using a Mann–Whitney-U for continuous data, and the Fisher exact test for categorical variables as appropriate.

#### Generic PDR to sounds

We analysed the variation of the pupil diameter in a time window of interest starting with the beginning of the baseline window and ending with the trial (− 500–3000 ms). For each participant, LSGS trials were compared to the mean baseline (− 500–0 ms before first sound onset) with a sample-by-sample Student t-test corrected for multiple comparisons with the false discovery rate (FDR) in time (*p* ≤ 0.05).

#### Regularity violations of PDR effects

Responses to the violation of auditory regularity were studied at 3 levels: (i) the local effect was defined as the PDR difference between local deviant (LDGD and LDGS) and local standard trials (LSGS and LSGD), (ii) the global effect was defined as the PDR difference between global deviant (GDLS and GDLD) and global standard trials (GSLD and GSLS), (iii) the “maximal violation effect” (MVE) was defined by the PDR difference between trials that were both global and local deviant (LDGD) and trials that were both local and global standard (LSGS).

In each trial, the average pupil diameter computed from the baseline window was subtracted from all data points. For each participant, we computed the mean PDR for each condition. For the group analysis, we then performed paired t-tests at each time point (≤ *p* 0.05 after correction in time for multiple comparisons with the FDR). To compute single-participant statistics, single-trial data were compared using a sample-by-sample Student t-test.

#### ERPs

For individual subject statistics, unpaired Welch’s tests were performed for each time sample. An effect was considered significant if it satisfied the following triple-threshold criterion *p* < 0.05 on a minimum of five consecutive samples (20 ms), on a minimum of 10 contiguous electrodes,during the expected time window of the corresponding ERP effect. For the local effect, an ERP was expected from 100 ms after the first sound onset to the end of epochs, whereas the time window ranged from 200 ms to end of epoch for the global effect^31^.

## Results

### Patients’ characteristics

Among the 42 DoC patients enrolled for the study, 7 were excluded because they were in a comatose state, and one because he was conscious but with limited interaction capabilities due to a severe Guillain-Barré syndrome that involved cranial nerves with no pupillary response to noise and a weak pupillary light reflex (EMCS scoring with recovery of functional communication). Ten additional patients were rejected because of bad pupillometric data quality (i.e., rejection of more than 75% of the trials, because of too many artefacts). The final sample consisted of 24 patients (12 females; median age 43 years (IQR: 28–56)). Ten patients were in the VS/UWS group (median CRS-R 5, range 3–7) and 14 in the MCS group (median CRS-R range 7–18). Neurological examination confirmed the presence of pupillary light reflex in both eyes of each of these patients. Demographic and clinical findings (reported in Table 1) did not differ between the two groups.

**Table 1:** Patients’ characteristics.

N	Patient	Sex	Age	Etiology	Delay from accident (in day)	CRS-R (total)	CRS-R (subscale)	Drugs affecting pupil	Mechanical Ventilation
1	VS	M	43	Anoxia	33	3	0/0/1/1/0/1	No	Yes
2	VS	F	73	Anoxia	30	3	0/0/2/0/0/1	No	Yes
3	VS	F	36	Stroke	104	7	1/2/2/1/0/1	No	Yes
4	VS	F	38	TBI	45	7	1/1/2/1/0/2	Scopolamine	Yes
5	VS	M	28	Anoxia	34	6	2/0/2/1/0/1	No	Yes
6	VS	M	62	SAH	46	6	1/1/2/1/0/1	No	Yes
7	VS	F	51	Anoxia	724	5	1/1/1/1/0/1	Scopolamine	No
8	VS	M	26	Anoxia	67	5	1/0/1/2/0/1	Phenobarbital	No
9	VS	F	36	Anoxia	27	5	0/0/2/1/0/2	No	Yes
10	VS	F	63	ICH	49	5	0/0/2/1/0/2	No	Yes
11	MCS	F	56	SAH	1880	9	1/3/2/1/0/2	Propericiazine	No
12	MCS	M	63	Stroke	89	5	0/3/0/0/0/2	No	Yes
13	MCS	M	17	Hypoglycemia	242	7	1/3/2/1/0/2	No	No
14	MCS	F	56	TBI	37	10	2/3/2/1/0/2	Venlafaxine	Yes
15	MCS	F	38	TBI	45	8	1/3/2/1/0/1	No	No
16	MCS	M	44	TBI	1215	10	1/3/2/2/0/2	Ipratropium, scopolamine	No
17	MCS	M	29	Anoxia	35	11	2/3/2/2/0/2	Paroxetine	Yes
18	MCS	M	19	TBI	49	8	0/0/5/1/0/2	No	No
19	MCS	F	22	Anoxia	418	9	1/3/1/1/0/3	No	No
20	MCS	M	76	Epilepsy	12	14	3/5/2/2/1/1	Phenobarbital	Yes
21	MCS	F	43	SAH	69	14	4/5/0/1/1/3	Scopalamine	No
22	MCS	F	19	TBI	31	14	3/3/4/2/0/2	No	Yes
23	MCS	M	49	ICH	41	13	2/3/3/2/1/2	No	No
24	MCS	M	54	Stroke	498	18	3/4/5/2/1/3	Scopolamine, tramadol	No

*TBI* traumatic brain injury, *SAH* subarachnoid hemorhage, *ICH* intra-cerebral haemorrhage, CRS-R subscales: auditory function scale/visual function scale/motor function scale/oro-motor/verbal function scale/communication scale/arousal scale—with a higher score meaning a better function.

### Data quality

Median number of rejected trials was 17% (IQR: 7–25%, see Supplementary material Table S1) and did not differ between MCS and VS patients (Mann–Whitney-U *p* = 0.2). Six patients (2 in a VS/UWS and 4 in a MCS) for which trials rejection rate was > 25% and < 75% were labeled as “poor quality pupillometry datasets”. ERP data were acquired with a median time of 1 day before the pupillometry (4 ERP datasets were acquired either the same day as pupillometry (N = 4), or before pupillometry (N = 13, min = 4 days) or after (N = 4, max = 5 days). ERPs were not available in one patient and did not meet data quality criterion (see M&M) because of too many artefacts due to agitation in one other patient, and 9 additional patients (3 VS and 6 MCS) were labeled as “poor quality EEG datasets “(i.e., automatic voltage rejection in more than 10% of electrodes in more than 70% of epochs).

### A non-specific generic auditory PDR was present in most patients

**Figure 2:**
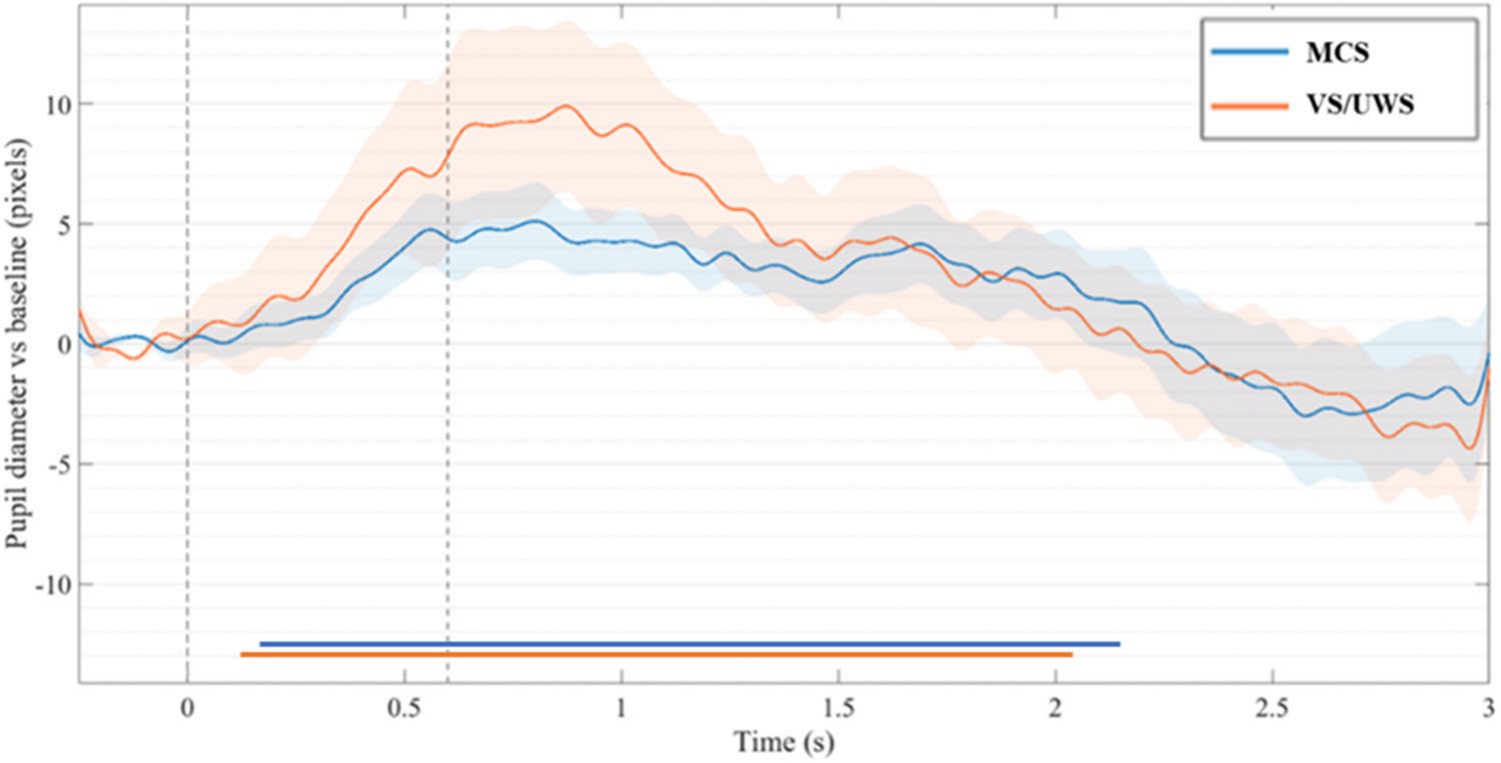
A non-specific pupil dilation response to sounds was observed both in the MCS and in the VS/UWS patients and did not differ between the two groups. Grand averages of pupil diameter (in pixels) are plotted over time (in seconds) in MCS (n = 14) and VS/UWS patients (n = 10). Grey dotted lines index first sound onset and fifth sound offset respectively. Temporal windows in which the pupil dilatation is significant from baseline are marked in with horizontal blue (MCS) and orange (VS/UWS) lines.

We first tested a non-specific generic PDR to auditory stimuli by computing the mean PDR in LSGS condition and testing PDR amplitude against baseline (t-test statistics significant at FDR corrected *p*-value ≤ 0.05; see M&M). At the group-level this generic and non-specific auditory PDR was present both in patients in a MCS and in patients in a VS/UWS (see Fig. 2). Baseline corrected auditory PDR did not differ significantly between MCS and VS/UWS patients (t-test statistics FDR corrected), although mean PDR was visually larger for the VS/UWS group than for the MCS group. We then ran the same analysis at the individual level: a generic auditory PDR was found significant in all patients (see “Sound” PDR column in Table 2), except for one VS/UWS patient and one MCS patient (note however that those patients had auditory ERP responses, see below).

**Table 2:** Behavioural, ERP, and PDR responses to sound and to violations of auditory regularities.

N patient		Beha viour	ERP					PDR					Outcome
		Startle	Quality	Sound	LDGD vs LSGS	Local effect	Global effect	Quality	Sound	LDGD vs LSGS	Local effect	Global effect	6 Months
1	VS	No	Poor	–	–	–	–	Correct	Yes	0.05	ns	ns	Death
2	VS	No	Poor	–	–	–	–	Correct	Yes	ns	ns	ns	Death
3	VS	Yes	Correct	Yes	Yes	Yes	No	Correct	Yes	ns	ns	ns	Conscious
4	VS	Yes	Poor	Yes	Yes	Yes	No	Correct	Yes	ns	ns	ns	Death
5	VS	Yes	Correct	Yes	No	No	No	Correct	Yes	ns	ns	ns	Death
6	VS	Yes	Correct	Yes	Yes	No	No	Correct	Yes	ns	ns	ns	MCS+
7	VS	Yes	Poor	Yes	Yes	Yes	No	Poor	Yes	ns	ns	ns	VS/UWS
8	VS	Yes	Correct	Yes	No	No	No	Poor	Yes	ns	ns	ns	VS/UWS
9	VS	No	Correct	Yes	Yes	Yes	No	correct	No	ns	ns	ns	VS/UWS
10	VS	No	Correct	Yes	No	Yes	No	Correct	Yes	ns	ns	ns	Death
11	MCS	Yes	Correct	Yes	Yes	Yes	No	Correct	Yes	ns	ns	ns	Na
12	MCS	No	Correct	Yes	Yes	No	Yes	Correct	No	ns	ns	ns	Death
13	MCS	Yes	Correct	Yes	Yes	Yes	Yes	Poor	Yes	ns	0.1	ns	Death
14	MCS	Yes	Poor	Yes	Yes	Yes	No	Correct	Yes	ns	ns	ns	Death
15	MCS	Yes	Poor	Yes	Yes	Yes	No	Correct	Yes	0.05	ns	ns	Death
16	MCS	Yes	Poor	Yes	No	No	No	correct	Yes	ns	ns	ns	MCS-
17	MCS	Yes	Poor	Yes	Yes	Yes	No	Poor	Yes	ns	ns	ns	MCS-
18	MCS	No	Poor	Yes	Yes	Yes	No	Correct	Yes	0.1	ns	0.1	Conscious
19	MCS	Yes	Poor	Yes	No	Yes	No	Poor	Yes	0.05	ns	ns	MCS-
20	MCS	Yes	Correct	Yes	Yes	Yes	No	Correct	Yes	0.05	ns	ns	Conscious
21	MCS	Yes	Correct	Yes	Yes	Yes	Yes	Poor	Yes	ns	ns	ns	Conscious
22	MCS	Yes	Correct	Yes	Yes	No	Yes	Correct	Yes	0.1	ns	ns	Conscious
23	MCS	Yes	Correct	Yes	No	No	No	Correct	Yes	ns	ns	ns	Conscious
24	MCS	Yes	Correct	Yes	Yes	Yes	Yes	Correct	Yes	0.05	ns	0.05	Conscious

LD: local deviant, LS: local standard, GS: global standard, GD: global deviant, local effect (LD vs LS), global effect (GD vs GS), “0.05” et “0.1” *p* < 0.05 or < 0.1 in a Student t-test contrasting standard and deviant trials at the individual scale. (“n.s” for non significative result, “Na “non available results).

### Pupil dilation response (PDR) to violations of auditory regularities

**Figure 3:**
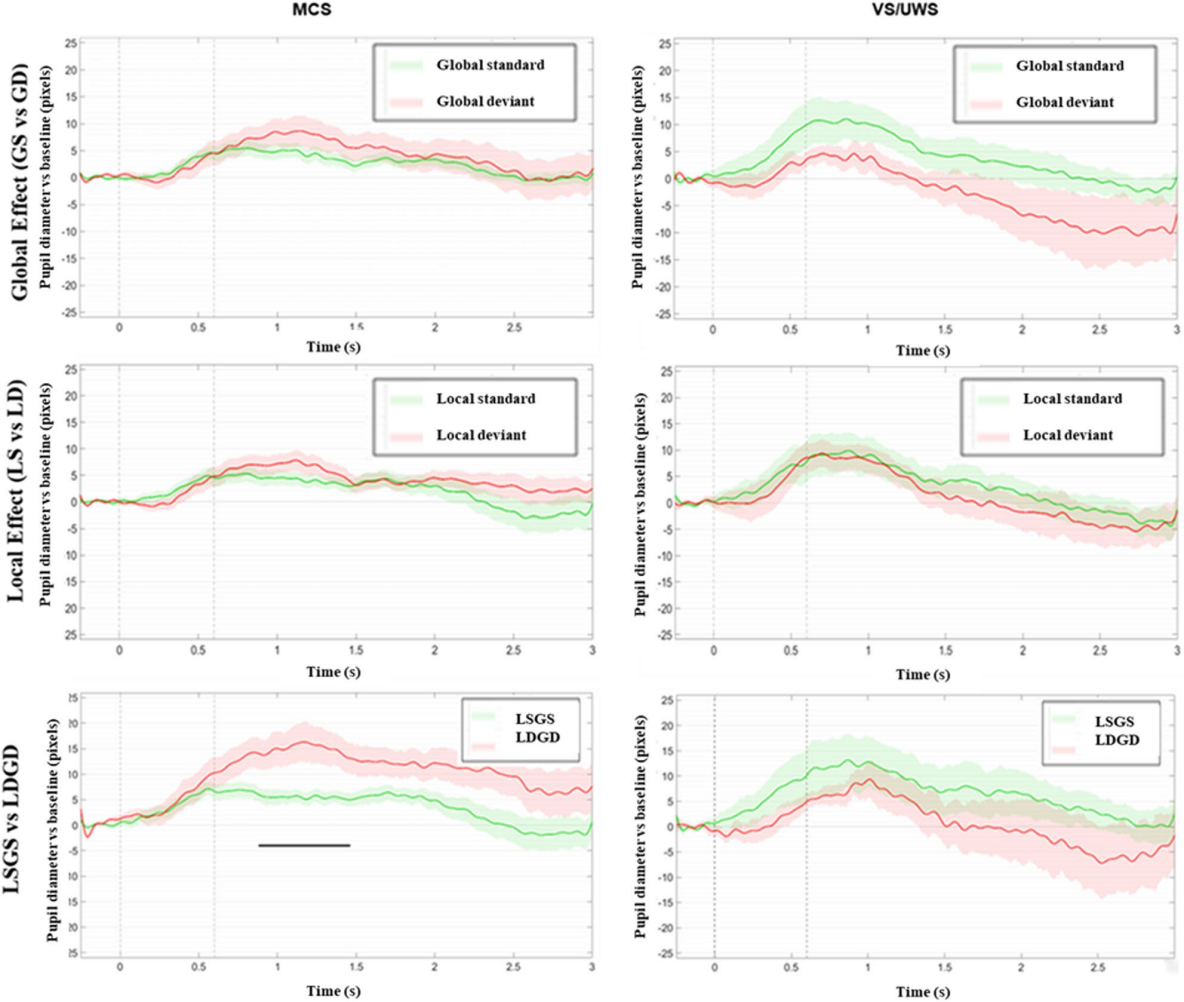
Local, global and maximal violation PDR effects for MCS and VS/UWS groups of DoC patients. Grand averages of pupil diameter (in pixels) are plotted over time (in seconds) in MCS (n = 14) and VS/UWS patients (n = 10) in an oddball paradigm. While the event-related pupil response was undistinguishable between LSGS (green curves) and LDGD (red curves) trials in the VS/UWS group, a significant (black horizontal segments) pupil dilation was observed in response to deviant trials in the MCS group. To distinguish conscious processing of inter-trials global regularity from non-conscious processing of intra-trials local regularity we contrast local standard trials to local deviant trials and global standard trials to global deviant trials and found no global or local effect in both groups. Grey dotted lines index first sound onset and fifth sound offset respectively.

At the group-level, we did not find significant local or global PDR effects, both in the whole population of patients and within each MCS and VS/UWS groups (t-test statistics significant at FDR corrected *p*-value, all *p*-values > 0.05, Fig. 3). Note that in VS/UWS patients although mean PDR was visually larger for the standard trials than for the deviant trials, this difference did not reach significance. In order to maximize the sensitivity of PDR, we finally contrasted the two maximally different conditions: LDGD vs LSGS trials. At the group level a significantly larger pupil dilation was observed after LDGD trials than after LSGS trials in MCS patients (FDR corrected *p* < 0.05; see Fig. 3 bottom panels). In contrast this effect was absent in VS/UWS patients. Besides, the PDR LDGD-LSGS difference was larger in MCS patients compared to VS/UWS patients during the 0.7–1.7 s time window interval (FDR corrected *p* < 0.05).

**Figure 4:**
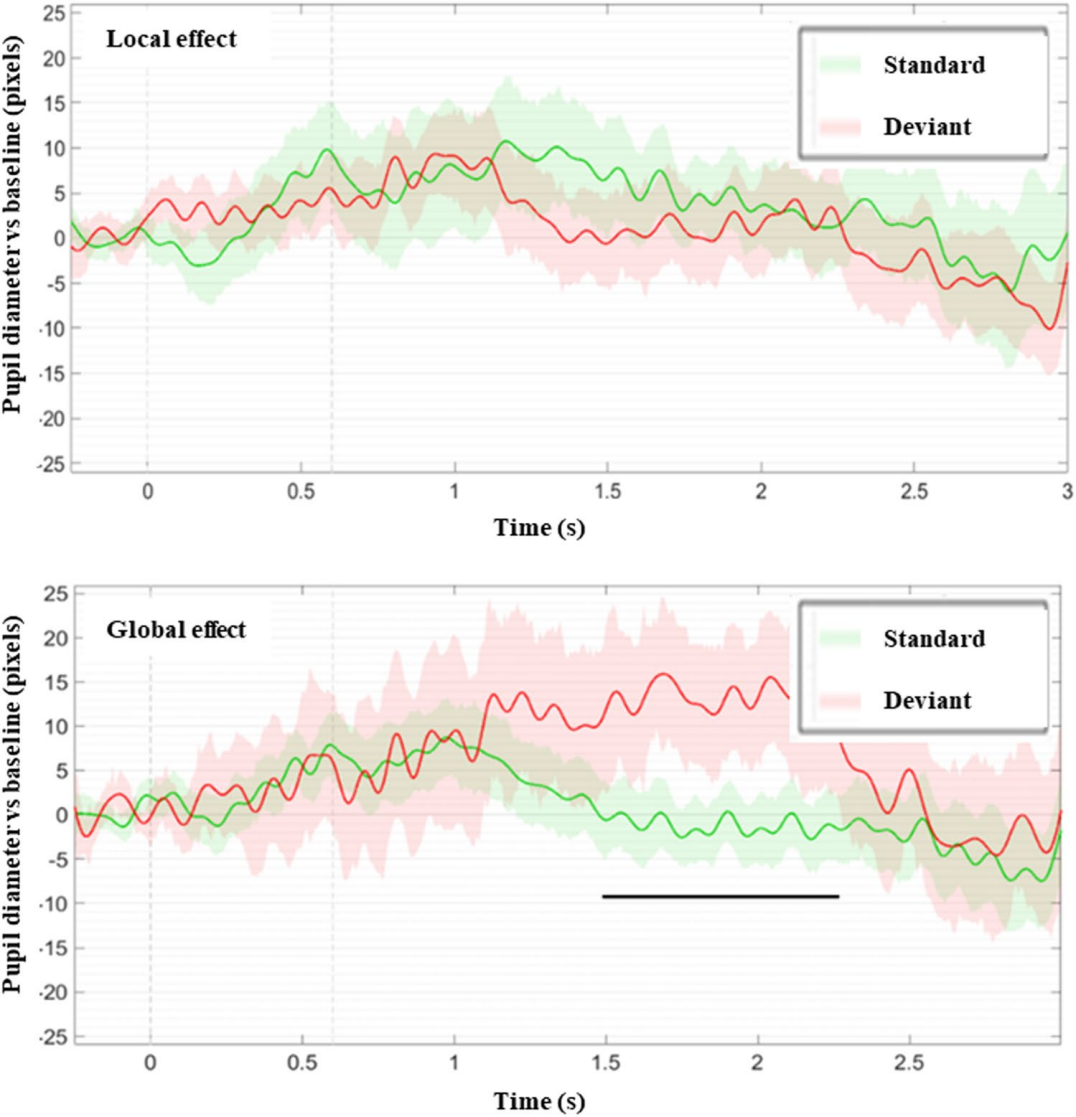
In MCS patient n°24 while the event-related pupil response was undistinguishable between local standard (green curves) and local deviant (red curves) trials, a global effect was observed: a significant sustained (black horizontal segment) pupil dilation was observed in response to global deviant trials. Grey dotted lines index first sound onset and fifth sound offset respectively. This patient regained consciousness 6 months later.

At the individual level, we followed the same approach. All but 2 patients showed a generic auditory PDR effect. As a quality check, these two patients did not show any significant local, global or LDGD/LSGS effects. No patient had a significant local effect, one patient showed a trend of a local effect (0.05 < *p* < 0.1; MCS patient #13) and did not regain consciousness. Only one patient showed a PDR global effect (MCS patient #24, Fig. 4, see Table 2), and another patient also in a MCS showed a trend of a global effect (0.05 < *p* < 0.1; MCS patient #18). Both patients regained consciousness at 6 months.

Finally, the [LSGS vs LDGD] contrast revealed a significant effect in 4 MCS patients and in 1 VS/UWS patient (see Table 2). Two additional MCS patients showed a trend for this effect (0.05 < *p* < 0.1). Note however that presence of [LSGS vs LDGD] effect (significant or with a trend) did not differ between MCS and VS/UWS patients in our small sample of patients (Fisher exact *p* = 0.17; sensibility to discriminate MCS from VS/UWS 43% (18–71); specificity 90% (56–100); PPV 86% (46–98); NPV 53% (41–65)).

Outcome data was available for 23/24 patients. Generic PDR effect to sounds was much too ubiquitous to be associated with any significant predictive value (*p* = 1). PDR Local (N = 1) and global (N = 2) effects were too rare to be correlated with any outcome in this limited sample of patients. Notably, the [LSGS vs LDGD] PDR effect showed a higher performance: 4/7 patients with a significant effect or a trend of an effect (*p* = 0.1) regained consciousness, versus 3/16 for patients who lacked this effect (Fisher exact *p* = 0.1; sensibility to predict recovery of consciousness: 57% (18–91), specificity 81% (54–96), PPV 57% (29–82), NPV 81% (64–91)).

### Event-related potentials

ERP results were available for 8/10 patients in a VS/UWS, and for all (14/14) patients in a MCS. Early cortical responses to sounds were observed in all patients (see “Sound” ERP column in Table 2), whereas a behavioural auditory startle was absent in 2/8 VS/UWS patients, and in 2/14 MCS patients (Table 2).

At the group level, a significant local effect and a significant LSGS vs LDGD effect were observed in the whole population of patients, as well as in MCS and in the VS/UWS groups (t-test statistics significant at FDR corrected *p*-value all *p*-values < 0.05). In contrast, a global effect was not observed, neither in the whole population of patients nor in MCS and VS/UWS groups (t-test statistics significant at FDR corrected *p*-value all *p*-values > 0.05).

At the individual level, a local effect was found significant in 10/14 MCS patients and in 5/8 VS/UWS patients (Fisher exact *p* = 0.4; sensibility 71% (42–92), specificity 38% (9–76); PPV: 67% (52–79); NPV: 43% (18–72)). Among these patients, 5 regained consciousness (Fisher exact *p* = 0.7, sensibility to predict recovery of consciousness 71% (29–96); specificity 36% (13–65); PPV 36 (23–51) %; NPV 71% (39–91)).

A global effect was found in 5 patients who were all in a MCS (Fisher exact *p* = 0.1; sensibility to discriminate MCS from VS/UWS 36% with 95% CI (13–65), specificity 100% (63–100), PPV 100%, NPV 43% (38–57)). Among the 5 patients with a global effect, 3 recovered consciousness at 6 months.

An ERP [LSGS vs LDGD] effect was found in 5/8 VS/UWS patients and in 11/14 MCS patients (Fisher exact *p* = 0.6; sensibility 79% (49–95), specificity 38% (9–76); PPV: 69% (55–80); NPV: 50% (21–79)). Among these patients, 5 regained consciousness (sensibility to predict recovery of consciousness (Fisher exact *p* = 0.6 ; 86% (42–79); specificity 36% (13–65); PPV 40 (29–52) %; NPV 83% (42–97)).

The two patients with a significant or a trend of a PDR global effect also had an EEG global effect. Among the patients with a significant or a trend [LSGS vs LDGD] PDR effect 5/6 patients also have an ERP [LSGS vs LDGD] effect. The presence of an ERP and a PDR global effect were not statically correlated (Fischer exact *p* = 0.4). The presence of an ERP and a PDR [LSGS vs LDGD] effects were not statically correlated either (Fischer exact *p* = 0.6).

## Discussion

In this study, we explored PDR in a population of DoC patients exposed to the local global auditory paradigm. At the group level, we found a generic PDR to sounds that was similar for MCS and VS/UWS groups, and we did not observe any significant local effect. These two results are in line with our previous report using the very same paradigm in healthy volunteers^39^. The preservation of generic auditory PDR response in VS/UWS patients is consistent with the previously reported early phasic pupillary dilation response corresponding to the automatic low-level processing of stimuli^18^. Note however that contrarily to our previous finding in conscious healthy volunteers^39^ we could not find a PDR global effect in groups of patients (neither across all patients nor in MCS or in VS/UWS groups). This negative result may stem from at least two factors that are not mutually exclusive. First, the global effect requires working memory resources and attentional engagement^31^, and is therefore very demanding cognitively. Our recent survey^33^ indeed confirmed that most patients showing a preserved ERP global effect were probably conscious (i.e., including patients with cognitive-motor dissociation^36^ during recording. Second, part of this negative result may also stem from signal-to-noise ratio issue, and in particular from poor data quality (i.e., resulting in numerous rejected trials), and the slow dynamics of PDR that forced us to record a lower number of trials, as compared with ERPs. Future methodological developments may improve this power issue, such as using deconvolution techniques using a fast event-related PDR paradigm as in fMRI^43^. This signal-to-noise ratio issue is made even more important when considering the difficult conditions of recording the ICU (i.e. including a lot of irrelevant auditory and light stimulation), in patients under various pharmacological treatments that may affect pupil dilation, and who frequently show eye blinks, head movements and other sources of artefacts. Finally, the head-mounted device used in the present study may have induced more artefacts than other eye-tracker devices.

Confronted to these limitations, we then defined a “maximal violation effect” by contrasting LSGS to LDGD trials in order to maximize differences in cognitive processing between, on the one hand, trials that respected both intra and inter-trial regularities, and on the other hand trials that violated both intra and inter-trial regularities. We could then detect a significant [LSGS vs LDGD] PDR effect for the group of MCS patients. Of particular interest, this effect was absent in the group of VS/UWS patients. Given that pupil dilation has been associated with conscious effortful processing, this finding suggests a richer cortical processing in MCS than in VS/UWS patients, as recently conceptualized under the acronym of cortically mediated state (CMS): MCS as defined by CRS-R criterion does not necessarily imply a residual consciousness but necessarily implies the existence of a CMS^6^.

We then applied the same approach at the individual level from generic auditory PDR to local, global and MVE effects. A generic auditory PDR was present in almost all patients except two VS/UWS patients and was therefore not a good predictor of diagnosis (i.e., MCS vs VS/UWS), or of consciousness recovery outcome. The only patient showing a significant PDR global effect was in MCS and recovered consciousness at 6 months.

Of special interest, presence of a significant individual [LSGS vs LDGD] PDR in 4 MCS patients and in 1 VS/UWS patient showed a trend of a predictive effect on consciousness recovery outcome. Concerning the VS/UWS patient with a significant LSGS vs LDGD] PDR effect, he died a few weeks after the evaluation and no evoked potentials were available. Besides we cannot determine whether his PDR effect reflected a defect in the specificity of the test, or demonstrated a richer state than clinically assumed. Many studies converge to show up to 15–20% of patients appear to be in a VS/UWS whereas their brain activity is suggestive of a MCS or even of a conscious state^2,44,45^.

ERPs were more sensitive than PDR to probe a global effect: a significant ERP MVE effect was found in 5 patients (4 MCS and 1 VS/UWS). Three of these patients recovered consciousness at 6 months. This last result tends to confirm the importance of artefacts and power issue (number of trials) that may have limited our ability to probe cognition with PDR in the current study.

Taken together, our results tend to suggest that bedside pupillometry and eye-tracking should still be considered as new approaches to explore consciousness and to predict recovery of consciousness in DoC patients. However, the current limitations we met at the individual level point to the necessity to solve several methodological issues. DoC patients blink rates are highly variable from one patient to another^46,47^ and are poorly controllable during the acquisition by the experiment in the absence of functional communication. When too abundant, and when other eye artefacts may impair PDR data (e.g., partial lid closures and sustained blepharospasms), PDR data may be unexploitable^48,49^. The current artefact correction techniques such as interpolation algorithms are not sufficient to overcome these limiting experimental conditions. Besides, patients must remain awake and non-agitated during the entire 6 min- paradigm to exploit pupillometric data and to be able to interpret them with high sensitivity to the current best conscious state. Here, among the 42 patients of the initial set, only 24 datasets (57%) were analyzable. Another difficulty stems from medications: in this study 41% of patients took medications such as opioids or scopolamine that probably influenced their pupillary function. Also, we cannot exclude that the pupillary response was artefacted by cognitive expectancy of the fifth sound. Indeed using the same paradigm, we previously measured an EEG contingent negative variation response (CNV) that corresponds to a slow anterior midline negative drift beginning from the onset of the first sound to the onset of the fifth sound^34,50,51^. In DoC patients the CNV was not more present in MCS or conscious state recordings than in VS recordings^34^. Another limitation inherent to pupillometry is the wide range of mental processes that can trigger pupillary dilations in the ICU environment such as pain, arousal fluctuations, displeasure and sudden environmental noise^52^.

To conclude, despite numerous technical challenges that remain to be overcome, these results open the way to a new window to probe residual cognition in DoC patients and by extension in non-communicating patients, at bedside, with cheap, non-invasive, relatively low-cost and non-constraining technology. Limitations pointed out by the present study should help to target the major directions of technological and methodological improvements, including an increase in the number of trials.

### Supplementary Information

Supplementary Table S1.

## Data Availability

The experimental datasets generated and analysed during the current study are available from the corresponding author upon reasonable request.
