# Supplementary material for: Pupil dilation response elicited by violations of auditory regularities is a promising but challenging approach to probe consciousness at the bedside
Source: Sci Rep. 2023 Nov 21;13:20331. doi: 10.1038/s41598-023-47806-1 (PMC10663629; doi:10.1038/s41598-023-47806-1)
Supplement: Supplementary file 1 — Supplementary Table S1. [file 41598_2023_47806_MOESM1_ESM.docx]

| **Patient** | **Quality** | **% of trial rejected** | **LSGS** | **LDGD** | **LDGS** | **LSGD** |
| --- | --- | --- | --- | --- | --- | --- |
| VS 1 | Correct | 4 | 26/28 | 11/10 | 30/30 | 10/10 |
| VS 2 | Correct | 3 | 24/24 | 12/13 | 25/26 | 12/12 |
| VS 3 | Correct | 10 | 33/34 | 8/8 | 25/26 | 6/12 |
| VS 4 | Correct | 17 | 26/26 | 12/12 | 22/30 | 5/10 |
| VS 5 | Correct | 23 | 20/30 | 6/10 | 27/32 | 9/9 |
| VS 6 | Correct | 10 | 25/26 | 8/12 | 25/28 | 11/11 |
| VS 7 | Poor | 36 | 18/28 | 5/11 | 25/34 | 4/8 |
| VS 8 | Poor | 32 | 20/27 | 9/11 | 18/32 | 7/9 |
| VS 9 | Correct | 1 | 26/26 | 11/12 | 32/32 | 9/9 |
| VS 10 | Correct | 16 | 24/28 | 10/11 | 25/26 | 6/12 |
| MCS-1 | Correct | 22 | 22/28 | 10/11 | 19/28 | 10/11 |
| MCS-2 | Correct | 3 | 27/28 | 11/11 | 32/32 | 8/9 |
| MCS-3 | Poor | 38 | 14/26 | 4/12 | 18/26 | 11/12 |
| MCS-4 | Correct | 5 | 25/28 | 10/11 | 30/30 | 10/10 |
| MCS-5 | Correct | 5 | 28/28 | 11/11 | 30/30 | 6/10 |
| MCS-6 | Correct | 13 | 24/28 | 9/11 | 25/28 | 10/11 |
| MCS-7 | poor | 27 | 24/32 | 7/9 | 14/22 | 11/14 |
| MCS-8 | correct | 7 | 21/22 | 10/11 | 24/25 | 9/11 |
| MCS-9 | poor | 47 | 15/28 | 6/11 | 13/28 | 7/11 |
| MCS+1 | correct | 21 | 29/30 | 8/10 | 23/34 | 5/8 |
| MCS+2 | poor | 35 | 21/28 | 6/11 | 16/28 | 8/11 |
| MCS+4 | correct | 17 | 22/28 | 8/11 | 32/34 | 5/8 |
| MCS+5 | correct | 22 | 25/30 | 6/10 | 22/28 | 9/11 |
| MCS+6 | correct | 24 | 20/32 | 6/9 | 25/28 | 8/9 |

***Supplementary Table 1 : Quality of pupillometric data.*** *Trials rejection rate with number of trials analysed for each condition / number of total trials for each condition (LD = Local Deviant ; LS = Local Standard, GS : Global Standard, GD : Global Deviant*
